# Restrained management of copper level enhances the antineoplastic activity of imatinib *in vitro* and *in vivo*

**DOI:** 10.1038/s41598-018-19410-1

**Published:** 2018-01-26

**Authors:** Iftekhar Hassan, Azmat Ali Khan, Shazia Aman, Wajhul Qamar, Hossam Ebaid, Jameel Al-Tamimi, Ibrahim M. Alhazza, Ahmed M. Rady

**Affiliations:** 1 Department of Zoology, College of Science, King Saud University, Riyadh, 11451 Saudi Arabia; 2 Pharmaceutical Biotechnology Laboratory, Department of Pharmaceutical Chemistry, College of Pharmacy, King Saud University, Riyadh, 11451 Saudi Arabia; 3 Department of Biochemistry, J N Medical College and Hospital, Aligarh Muslim University, Aligarh, India; 4 Biological Unit, Central Laboratory, Research Center, College of Pharmacy, King Saud University, Riyadh, 11451 Saudi Arabia; 5 Department of Pharmacology and Toxicology, College of Pharmacy, King Saud University, Riyadh, 11451 Saudi Arabia

## Abstract

The present study was designed to investigate if elevated copper level can be targeted to enhance the efficacy of a significant anticancer drug, imatinib (ITB). The antineoplastic activity of this drug was assessed in the HepG2, HEK-293, MCF-7 and MDA-MD-231 cells targeting elevated copper level as their common drug target. The cell lines were treated with the different doses of copper chloride (Cu II) and disulfiram (DSF) alone as well as in their combinations with the drug for 24 h in standard culture medium and conditions. The treated cells were subjected to various assays including MTT, PARP, p-53, caspase-7, caspase-3, LDH and single cell electrophoresis. The study shows that DSF and Cu (II) synergizes the anticancer activity of ITB to a significant extent in a dose-specific way as evidenced by the combinations treated groups. Furthermore, the same treatment strategy was employed in cancer-induced rats in which the combinations of ITB-DSF and ITB-Cu II showed enhanced antineoplastic activity as compared to ITB alone. However, DSF was more effective than Cu (II) as an adjuvant to the drug. Hence, restrained manipulation of copper level in tumor cells can orchestrate the redox and molecular dispositions inside the cells favoring the induction of apoptosis.

## Introduction

Cancer is the second largest life-threatening disease in the world after cardiovascular diseases^1^. The disease arises from genetic mutation either by inheritance or by acquiring during one’s lifetime. Such genetic mutations trigger proto-oncogenes into oncogenes consequently leading to cancer. Although our body is well-equipped with various types of antitumor machinery including tumor suppressor gene p-53, still many of the severe mutations persist in the cells turning them into cancerous ones. The latest data suggest that rate of cancer incidence in the world will increase the burden of the disease by many folds shortly^2^. The treatment modalities available for cancer treatment today are based on chemotherapy, radiotherapy, adjuvant therapy, hormonal therapy, catalytic therapy and gene therapy^3–5^. The effect of these treatment modalities are either short-lived or accompanies with serious side effects that further exacerbate the overall suffering of the patients^6^. One of the important hurdles in the way of cancer treatment is a lack of established common denominator of the disease that can be the possible drug targets. Many scientists have suggested various anticancer treatment strategies that might pave pathways for a fruitful breakthrough in future^7,8^. Recently, plenty of researchers applauds elevated endogenous copper level as one of the most prominent hallmarks in the cancerous cells^6,9^.

Copper is believed to be one of the essential trace metals beside iron in our body. Its vital role in catalysis of various metabolic enzymes, nerve induction, and angiogenesis along with assisting all iron mediating biological actions and boosting the immunity are well documented^10,11^. Intriguingly, the level of copper mounts by many folds in most of the types of cancer irrespective of the origin^12^. It is assumed that the increased copper level facilitates angiogenesis in the cancer cells^13^ that is one of the reasons for chemotherapy resistance in the cells. Besides, copper being an active transition element, can act as pro-oxidant or antioxidant depending on the concurrent cellular redox status^6^. Tapping this dual nature of this metal for killing the cancer cells has been a hotspot for oncological research for the last three decades^14–17^. However, many investigators believe that elevated copper level can attribute to the resistance against chemotherapy in the cancer cells^18^. Hence, the copper chelating agents can enhance the efficacy of the anticancer drugs under chemotherapy^19,20^. The clinically approved copper chelating agents (Disulfiram and Tetrathiomolybdate) against many diseases including Wilson’s disease and Alzheimer’s disease are under extensive oncological research nowadays. Furthermore, employing copper chelators is a very promising anticancer strategy to enhance target specificity and efficacy of the contemporary antineoplastic agents^21^.

## Hypothesis

The level of endogenous copper if manipulated in such a way that can orchestrate the cellular redox status at the optimum level; it can trigger many vital pro-apoptotic proteins facilitating the conditions for induction of programmed cell death. It can consequently compel the cancer cells to undergo apoptosis to a greater extent that is considered as the most preferred way to kill the such cells.

The present investigation was aimed at testifying our hypothesis on the cancer cell lines followed by its confirmation *in vivo*. This study was designed to investigate the elevated level of copper as drug- target to enhance the antineoplastic activity of ITB. At the same time, we wanted to confirm if supplementation of copper in excess or their deprivation by administration of disulfiram (copper chelating agent) can improve the chemotherapeutic potential of the drug^22^. For this, we tested our hypothesis on chemically induced hepatocarcinoma rat models under in vivo studies.

## Results

### *In vitro* studies

#### MTT assay

**Figure 1:**
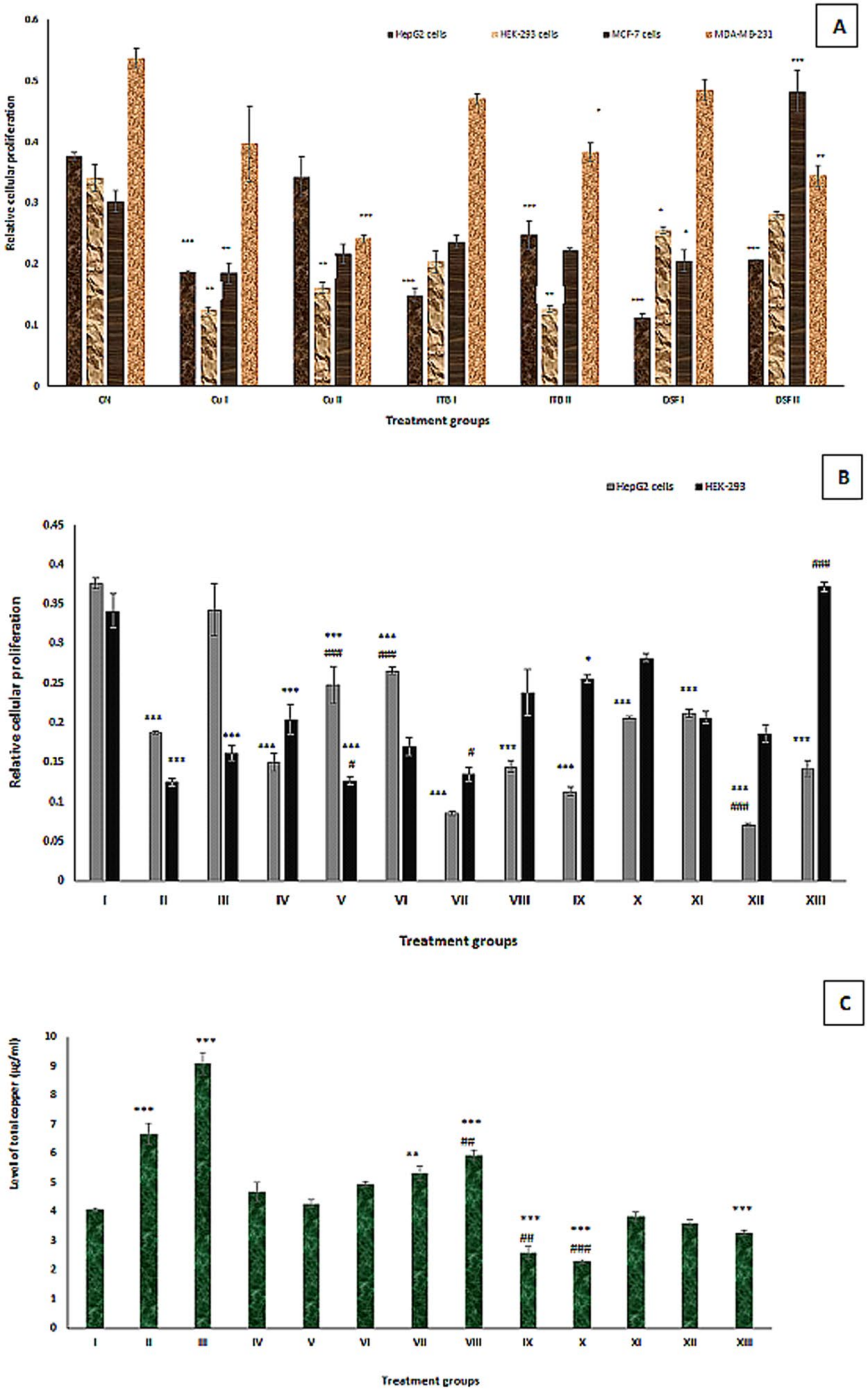
(**A**) MTT assay of four cell lines- HepG2, HEK-293, MCF-7 and MDA-MB-231 after treatment with copper chloride (CuCl_2_), imatinib (ITB) and disulfiram (DSF) for 24 h at two different doses. *Indicates values statistically significant from the control after selecting P < 0.05 as the level of significance during statistical analysis of the date. (**B**) MTT assay of two cell line- HepG2 and HEK-293 after treatment for 24 h with copper chloride (CuCl_2_), imatinib (ITB), disulfiram (DSF) and the combinations of imatinib with Cu and DSF. The values marked with *indicate statistically significant from the control while the values marked with #indicate statistically significant from the group IV after selecting P < 0.05 as the level of significance during statistical analysis of the date. (**C**) Showing the total intracellular copper level in the HepG2 cells treated with the test chemicals. The values marked with *indicate statistically significant from the control (group I) while the values marked with #indicate statistically significant from the group IV after selecting P < 0.05 as the level of significance during statistical analysis of the date.

Effect of test chemicals on cell viability in the cell lines: All the test chemicals-ITB, Cu (II) and DSF were tested on MCF-7, MBA-MD-231, HepG2 and HEK-293 cell lines by MTT assay (Fig. 1A). We found that the proposed compounds were not effective with the breast cancer cell lines, so both cell lines were not included in further studies under the present investigation.

The IC_50_ is a reliable parameter to assess cell viability and extent of cytotoxicity after treatment of any test compound in the cell line based study. In the present study, MTT assay of the test chemicals was conducted on the cell lines-HEK-293 and HepG2 with their respective IC_50_ (Table 1). It was observed that ITB, Cu (II) and DSF were more effective in the liver cell line as compared to the kidney cell line.

**Table 1:** Showing IC_50_ of copper chloride (CuCl2), imatinib (ITB) and disulfiram (DSF) in HEK-293 and HepG2 cells expressed in mean ± SEM.

Cell line	Test chemical	IC_50_ (in mM) after 24 h Mean ± SEM
HEK-293	Cu(II)	13.01 ± 0.42
	ITB	12.52 ± 0.58
	DSF	Not observed
HepG2	Cu(II)	10.52 ± 0.38
	ITB	8.06 ± 0.51
	DSF	10.05 ± 0.76

Effect of Cu (II) and DSF on antineoplastic activity of ITB in HEK-293 cells: In the present study, group II and III showed a decrease in proliferation by 99.96% and 52.78% in comparison to the control, group I. Moreover, Cu (II) exerted a significant effect on anticancer activity ITB in the kidney cells. Group VI demonstrated an increase in the proliferation by 17.15% with respect to group IV while group VII and VIII showed a dip in the proliferation by 33.82% and 15.15% respectively (Fig. 1B).

Furthermore, group IX and X exhibited a decline in the cellular proliferation by 24.92% and 17.30% with respect to the control. In the case of combination groups, group XI and XIII showed an increase in cellular proliferation by 0.98% and 82.35% respectively as compared to group IV although group XII demonstrated a decline in cellular growth by 8.82% against ITB alone treated group IV (Fig. 1B).

**Figure 2:**
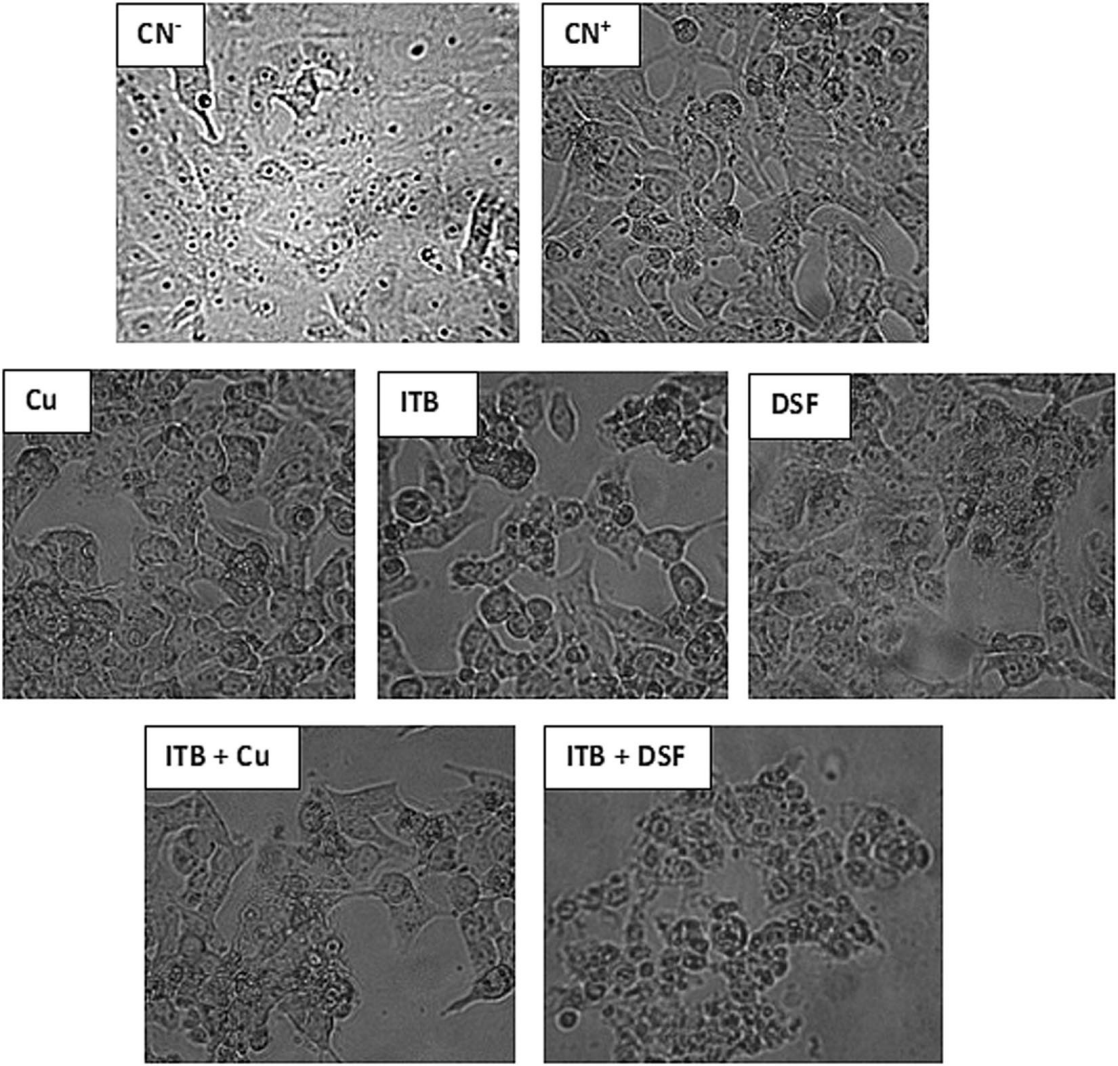
Showing pictures of main groups of HepG2 cells after treatment with the test chemicals for 24 hours. The groups showed in the figure are: Control negative (CN^−^); Control positive (CN^+^); Copper treated (Cu); ITB treated; DSF treated; ITB with copper (ITB + Cu); ITB with DSF (ITB + DSF). The groups were snapped from the culture plate by inverted microscope (Nikon, Japan) at 40X.

**Effect of Cu (II) and DSF on antineoplastic activity of ITB in HepG2 cells**: The treatment of Cu (II) in the HepG2 cells led to declining the cellular proliferation by 50.26% and 8.77% at the dose of 10 µl and 20 µl as evidenced by group II and III respectively with respect to the control. ITB treated, group IV and V showed an increase in cell death by 60.10% and 34.04% as compared to the control. Interestingly, group VI showed an increase in cell proliferation by 77.33%; however, group VII and VIII demonstrated killing of the cells by 43.33% and 4% respectively as compared to the group IV (Figs 1B and 2).

On the other hand, DSF treated group IX, and X exhibited a decrease in cell proliferation by 69.94% and 45.21% as compared to the control. However, group XI displayed an increase in the cell growth by 41.33% while the group XII and XIII showed a decrease in the cellular proliferation by 52.66% and 5.33% respectively with reference to group IV (Figs 1B and 2).

#### Effect on level of the total intracellular copper post-treatment with CuCl_2_, ITB, and DSF

Copper treated groups II and III showed a dose-dependent increase in intracellular copper level by 64.28% and 123.15% while group IV and V demonstrated the increased level of the metal by 15.27% and 5.42% respectively as compared to the control. However, the groups-VI, VII, and VIII exhibited a dose-dependent increase of the metal by 22.16%, 30.78% and 46.55%. In contrary, DSF treated groups-IX and X showed a decrease in the copper level by 36.20% and 43.59%. However, group XI, XII and XIII demonstrated a dose-dependent reduction of the metal with respect the control (Fig. 1C).

#### Effect of Cu (II) and DSF on the activity of p-53 (S1) in HepG2 cells treated with ITB

**Figure 3:**
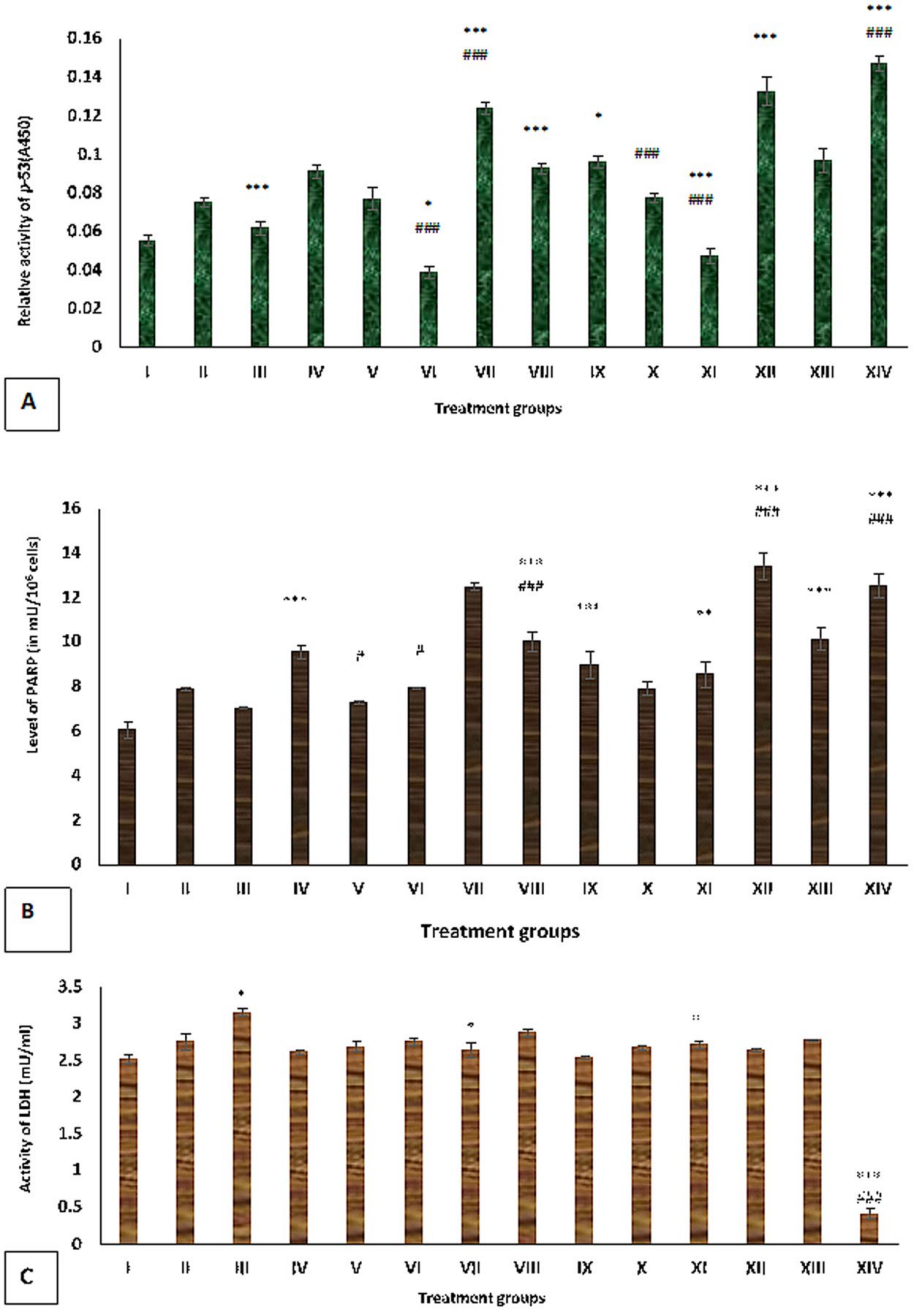
(**A**) Showing relative values of p-53 in the different treatment groups of HepG2 cells. The values marked with *indicate statistically significant from the control (group I) while the values marked with #indicate statistically significant from the group IV after selecting P < 0.05 as the level of significance during statistical analysis of the date. (**B**) Showing the level of Poly (ADP-ribose) polymerase (PARP) in the different groups of treated HepG2 cells expressed in mU/10^6^ cells. The values marked with *indicate statistically significant from the control (group I) while the values marked with #indicate statistically significant from the group IV after selecting P < 0.05 as the level of significance during statistical analysis of the date. (**C**) Showing activity of Lactate Dehydrogenase (LDH) in the different groups of treated HepG2 cells expressed in mU per ml of the cell supernatant. The values marked with *indicate statistically significant from the control (group I) while the values marked with #indicate statistically significant from the group IV after selecting P < 0.05 as the level of significance during statistical analysis of the date.

ITB treated groups- IV and V revealed an increase in p-53 activity by 65.45% and 40% while group II and III showed elevation in its activity by 36.36% and 10.90% with reference to the control. Group VI exhibited a decline in its activity by 57.14% while group VII and VIII showed 36.26% and 1.09% of the increase in the level of p-53 as compared to group IV (Fig. 3A).

Furthermore, the cells treated with DSF at 5 µl and 10 µl doses demonstrated an elevation in p-53 activity by 74.54% and 40% with respect to the control as shown by groups- IX and X. However, the group XI treated with a combination of ITB and DSF showed a decrease in the activity by 48.35% while the groups- XII and XIII displayed enhancement in its activity by 45.05% and 6.59% as compared to the group IV. The values of p-53 activity in group VII and XII were near to the healthy liver cells, group XIV (Fig. 3A).

#### Effect of Cu (II) and DSF on PARP activity in HepG2 cells treated with ITB

The level of PARP after treatment with Cu at 10 µl and 20 µl was increased by 30.52% and 16.17% as shown by group II and III. The ITB-treated group IV and V showed an increase in its level by 57.42% and 20.62% in comparison to the control, group I. However, the combination treated group VI demonstrated a decrease in its activity by 16.77% while group VII and VIII exhibited upregulation of PARP activity by 30.92% and 5.03% with respect to group IV (Fig. 3B).

Besides, DSF-treated groups- IX and X displayed the increase in the PARP level by 48.02% and 30.69% with respect to the control. Among the combinations, group XI showed a decrease in its level by 10.48% while group XII and XIII showed an increase in the level by 40.56% and 6.39% as compared to group IV. The values of PARP activity in group VII and XII were comparable to that of group XIV.

#### Effect of Cu (II) and DSF on lactate dehydrogenase (LDH) activity in HepG2 cells treated with ITB

It is well accepted among the oncologists that LDH is an important parameter to assess the extent of necrosis in cell line-based studies. In the present investigation, group II and III showed an increase in LDH activity by 9.12% and 25% while group IV and V demonstrated the enhancement of its activity by 3.57% and 6.35% with respect to the control. The combination groups- VI, VII, and VIII displayed an increase in the activity by 5.36%, 1.53% and 10.34% with respect to group IV (Fig. 3C).

Also, DSF treated group IX, and X exhibited an increase in the activity of LDH by 0.79% and 5.95% as compared to the control while the combination treated groups-XI, XII and XIII showed an increase in its activity by 3.69%, 1.15% and 6.13% with respect to group IV. However, the group XIV showed very negligible activity of this enzyme (Fig. 3C).

#### Effect of Cu (II) and DSF on caspase-7 activity in HepG2 cells treated with ITB

**Figure 4:**
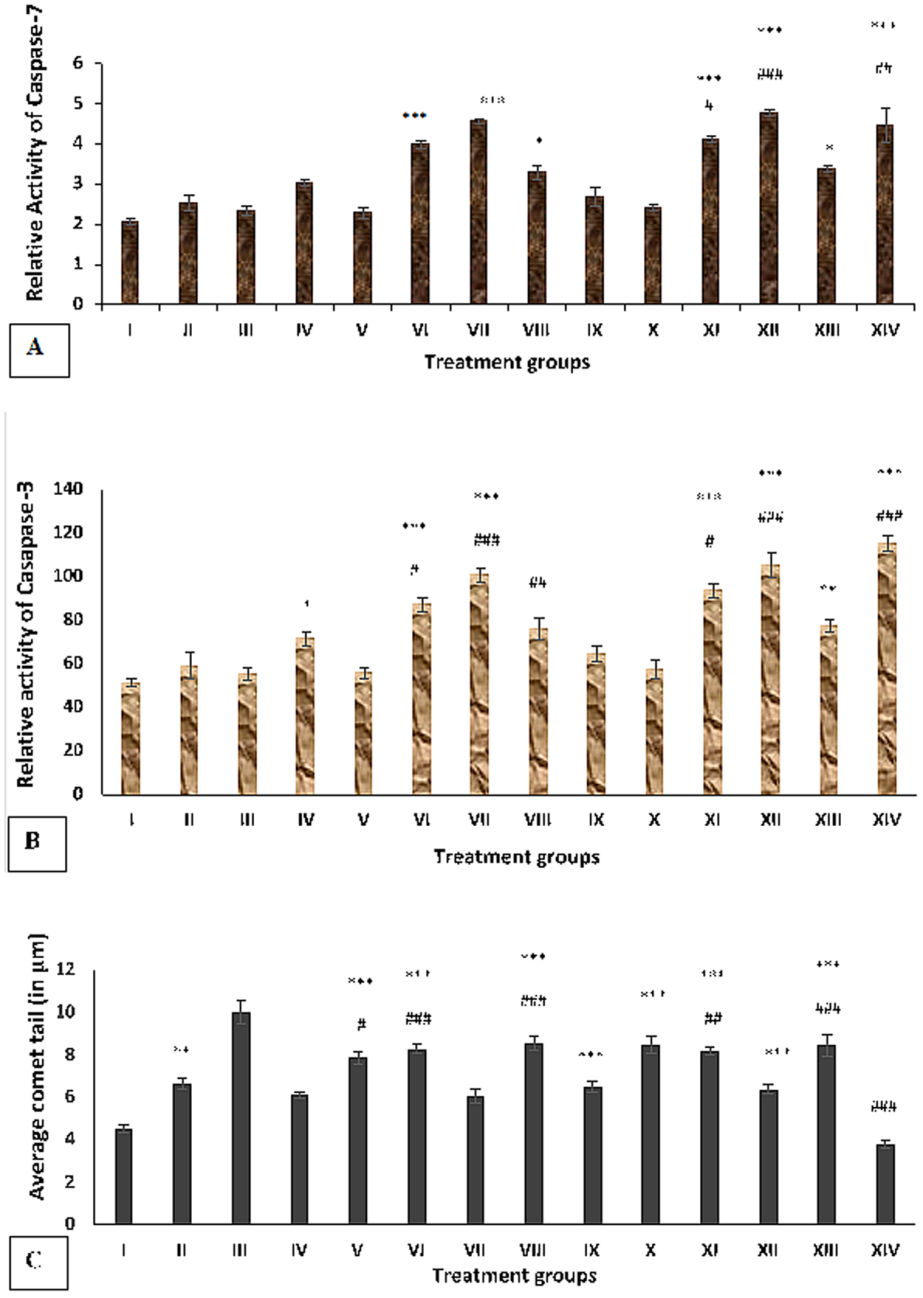
(**A**) Showing relative activity of caspase-7 in the different groups of treated HepG2 cells. The values marked with *indicate statistically significant from the control (group I) while the values marked with #indicate statistically significant from the group IV after selecting P < 0.05 as the level of significance during statistical analysis of the date. (**B**) Showing relative activity of caspase-3 in the different groups of treated HepG2 cells. The values marked with *indicate statistically significant from the control (group I) while the values marked with #indicate statistically significant from the group IV after selecting P < 0.05 as the level of significance during statistical analysis of the date. (**C**) Showing the average comet tail-length (in µm) in the different groups of treated HepG2 cells. The values marked with *indicate statistically significant from the control (group I) while the values marked with #indicate statistically significant from the group IV after selecting P < 0.05 as the level of significance during statistical analysis of the date.

In the present study, the activity of caspase-7 was assessed for confirmation of apoptosis progression. Group II and III showed its enhanced activity by 22.33% and 13.10% while group IV and V demonstrated an increase in the activity by 46.17% and 10.68% as compared to the control, group I. However, the combination treated groups-VI, VII and VIII exhibited an increase in its activity by 30.96%, 51.49%, and 8.97% respectively with respect to group IV (Fig. 4A).

In parallel, DSF treated group IX and X displayed an increase in the activity by 30.58% and 16.99% with respect to the control while the combination groups–XI, XII and XIII showed rise e in the activity by 36.54%, 58.80%, and 11.96% respectively as compared to group IV. The activity of both groups-VII and XII was quite comparable to that of group XIV (Fig. 4A).

#### Effect of Cu (II) and DSF on caspase-3 activity in HepG2 cells treated with ITB

After assessment of caspase-7, the activity of caspase-3 was the logical aim of the study. In the present investigation, group II and III was observed to have elevation in caspase-3 activity by 15.51% and 8.29% while group IV and V showed enhancement in its activity by 39.31% and 8.82% as compared to the control, group I. Group VI, VII, and VIII exhibited an increase in the activity by 21.96%, 41.31% and 9.57% with respect to group IV (Fig. 4B).

Moreover, DSF-treated groups- IX and X on the other hand, exhibited an increase in the activity by 25.36% and 12.45% as compared to the control. Hitherto, the combination treated groups-XI, XII and XIII demonstrated a rise in the activity by 30.89%, 47.62% and 8.41% with respect to group IV. The activity of both groups-VII and XII was quite close to that of group XIV (Fig. 4B).

#### Effect of Cu (II) and DSF on comet assay of HepG2 cells treated with ITB

**Figure 5:**
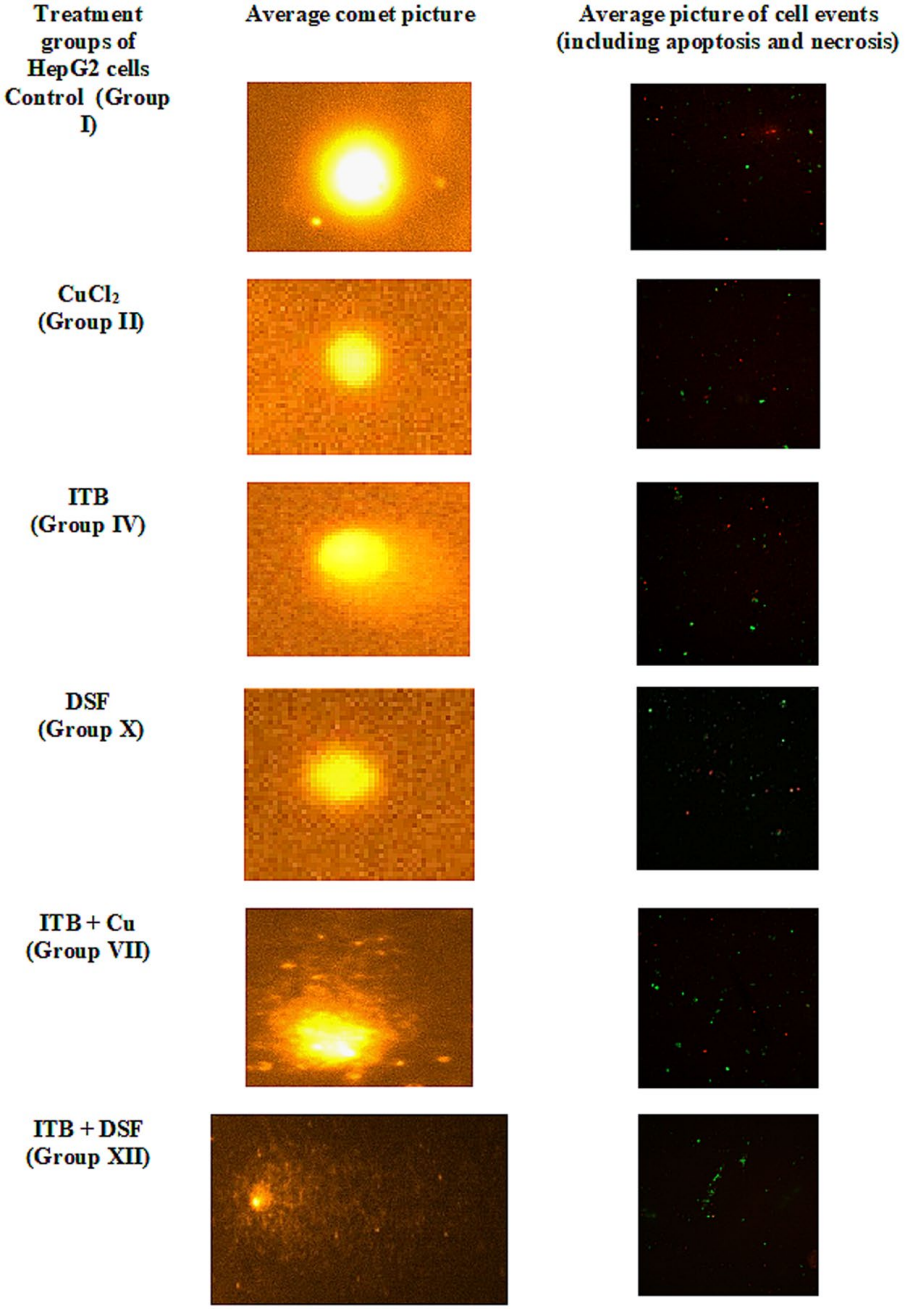
(**A**) Showing average picture of comet from major indicated groups. All pictures were taken at 100X with digital camera (Andor Zyla, UK) attached to the fluorescent microscope (Leica DM2500, Germany). (**B**) Showing the cells undergoing necrosis (red color), apoptosis (green color) for the major groups. The cells were stained with Propidium iodide and Alexa 488 on poly L-lysine coated microscope slides that were analyzed by confocal microscopy (Carl Zeiss, Germany) at 100X.

Comet assay is an important technique for assessment of damage to nuclear DNA that can also be indicative of the mode of cell death. In the present study, group II and group III exhibited increase in tail length by 47.24% and 120.53% while group IV and group V showed increase by 34.87% and 73.95% with respect to the control, group I. The combination groups- VI and VIII demonstrated increase in the tail length by 35.67% and 39.44% while group VII showed 0.49% of the decrease in the parameter. DSF treated groups- IX and X exhibited an increase in tail length by 43.70% and 87.42% while the combination groups- XI, XII and XIII displayed an increase in the tail length by 33.71%, 4.74% and 38.46% as compared to group IV respectively (Figs 4C and 5).

#### Confocal microscopy

The microscopy of the treated cells on slides revealed apoptotic cells in green color while the necrotic cells were observed as in red color. The group VI and XII showed more cells undergoing apoptosis as compared to the control (Fig. 5).

#### Flow cytometry

**Figure 6:**
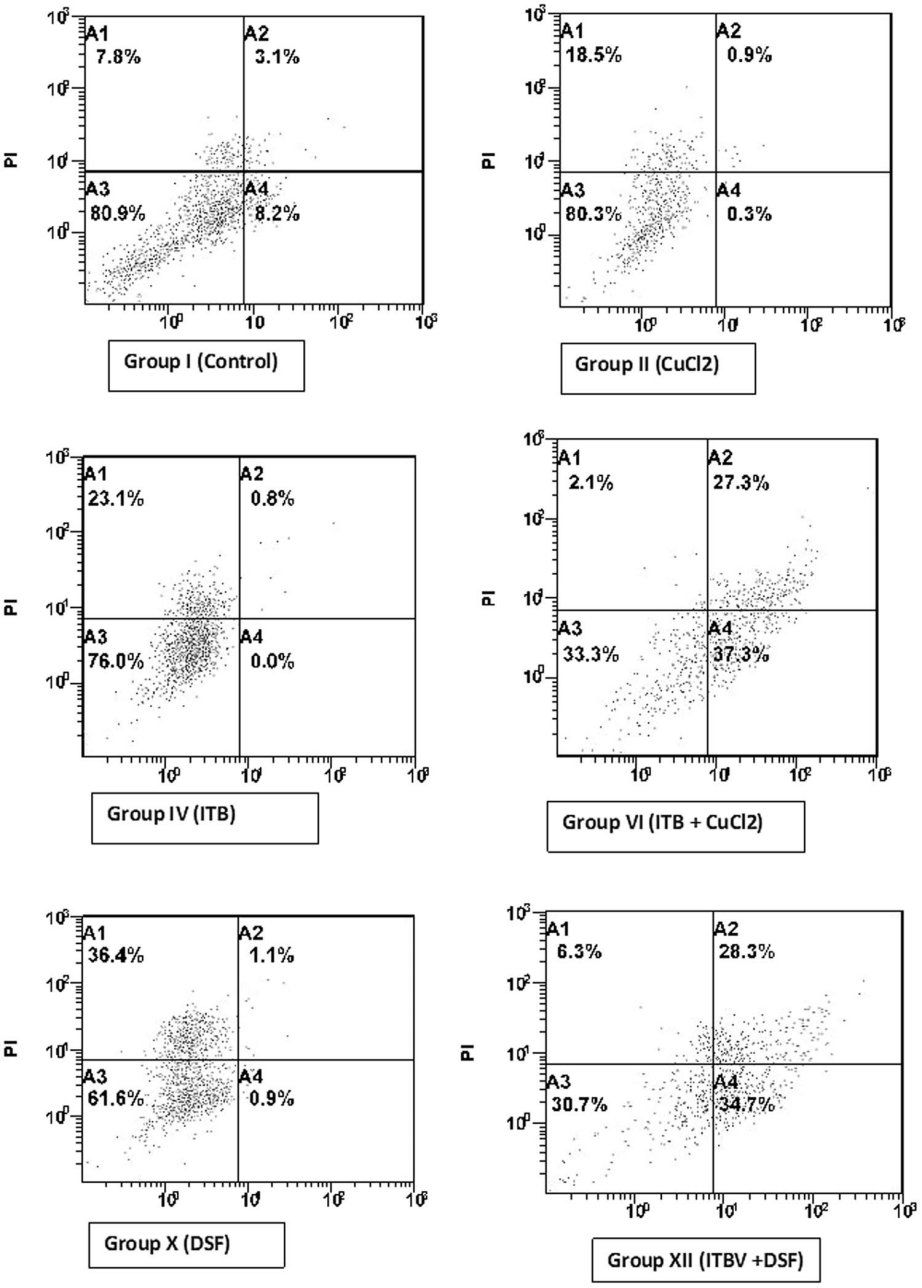
Showing the FACS of treated cells of the significant groups undergoing pre-apoptosis, apoptosis, and necrosis in quadrant A4, A2 and A1 as compared the respective control in quadrant A3.

The FACS of the treated cells demonstrated that Cu and DSF both elevated the antineoplastic activity of ITB as the cells undergoing apoptosis were significantly higher in group VI and group XII concerning the control (Fig. 6).

### *In vivo* studies

#### Effect on toxic burden in serum samples

**Figure 7:**
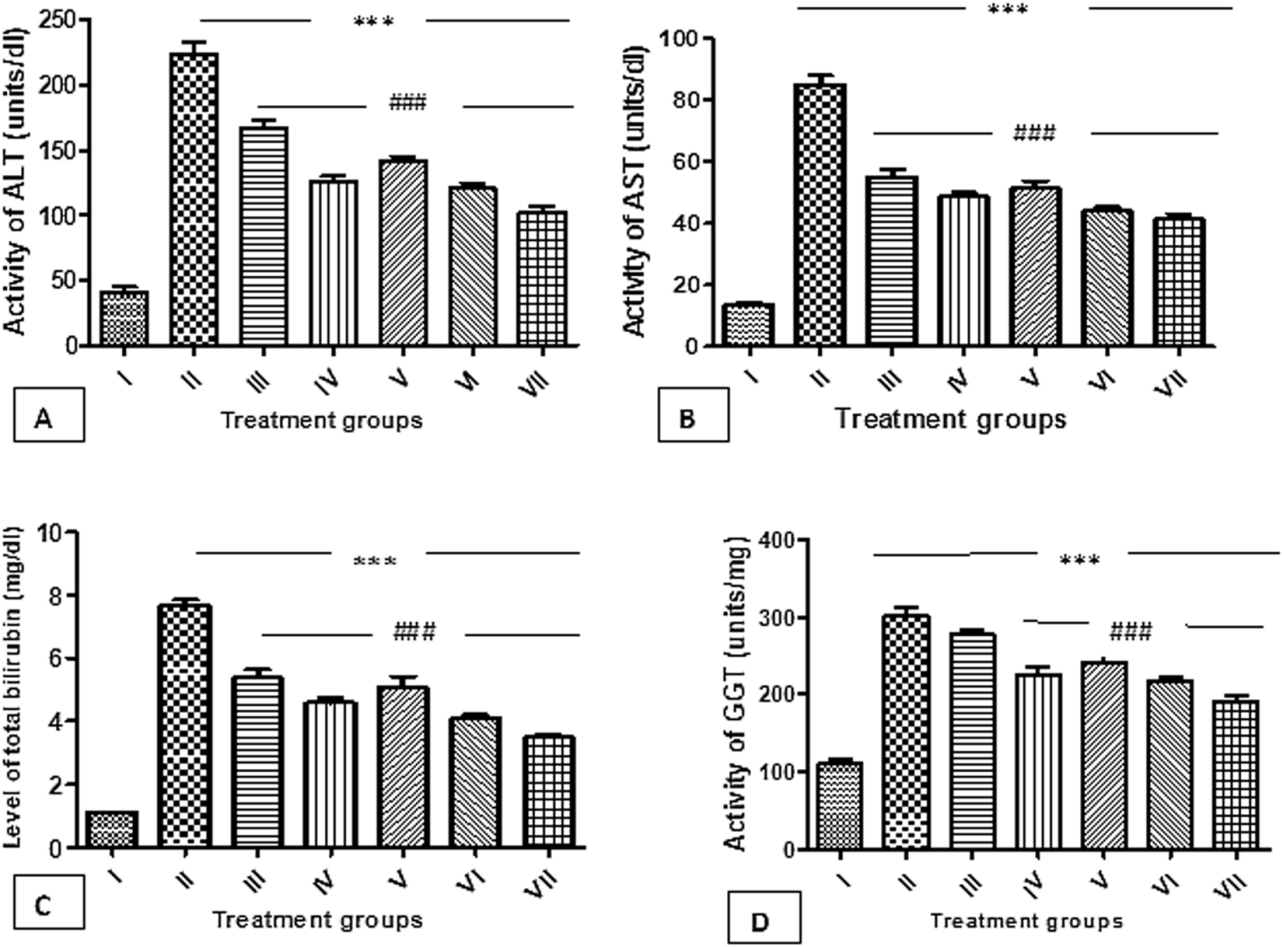
(**A**) Showing activity of ALT in the serum samples of different animal groups. *Indicates statistically significant from group I while #indicates statistically significant from group II. (**B**) Showing activity of AST in the serum samples of different animal groups. *Indicates statistically significant from group I while #indicates statistically significant from group II. (**C**) Showing level of total bilirubin in the serum samples of different animal groups. *Indicates statistically significant from group I while #indicates statistically significant from group II. (**D**) Showing activity of GGT in the serum samples of different animal groups. *Indicates statistically significant from group I while #indicates statistically significant from group II.

**Alanine transaminase (ALT)**: The activity of this enzyme was found to be increased by 451.11% in group II as compared to the control normal, group I. Group III, IV and V showed a decrease in its activity by 25.41%, 46.57%, and 34.31% respectively with respect to group II. However, the combination groups-VI and VII demonstrated a decline in its activity by 48.18% and 52.23% as compared to the group II (Fig. 7A)

**Aspartate transaminase (AST):** The effect of the treatment of the test chemicals showed a similar pattern as of ALT. Group II exhibited an extensive enhancement in its activity by 522.58% as compared to group I. Group III, IV and V displayed dip in its activity by 35.18%, 42.71%, and 39.45%; however, the groups-VI and VII demonstrated a sharp decline in the activity by 47.99% and 51.31% respectively in comparison to group II (Fig. 7B).

#### Total serum bilirubin

Group II demonstrated an increase in its level by 590.09% as compared to group I. Group III, IV and V exhibited a decrease in its level by 29.76%, 40.20% and 33.81% with reference to group II. However, group VI and VII showed a decline in its level by 46.73% and 54.57% as compared to group II respectively (Fig. 7C).

**γ- glutamyl transferase (GGT):** Group II showed the enhanced level of this enzyme by 170.36% concerning group I. Group III, IV and V demonstrated a decrease in its level by 8.02%, 24.85% and 20.04% as compared to group II. Hitherto, group VI and VII displayed a decline in its activity by 28.14% and 36.45% respectively in comparison to group II (Fig. 7D).

#### Effect on the level of Bax-protein

**Figure 8:**
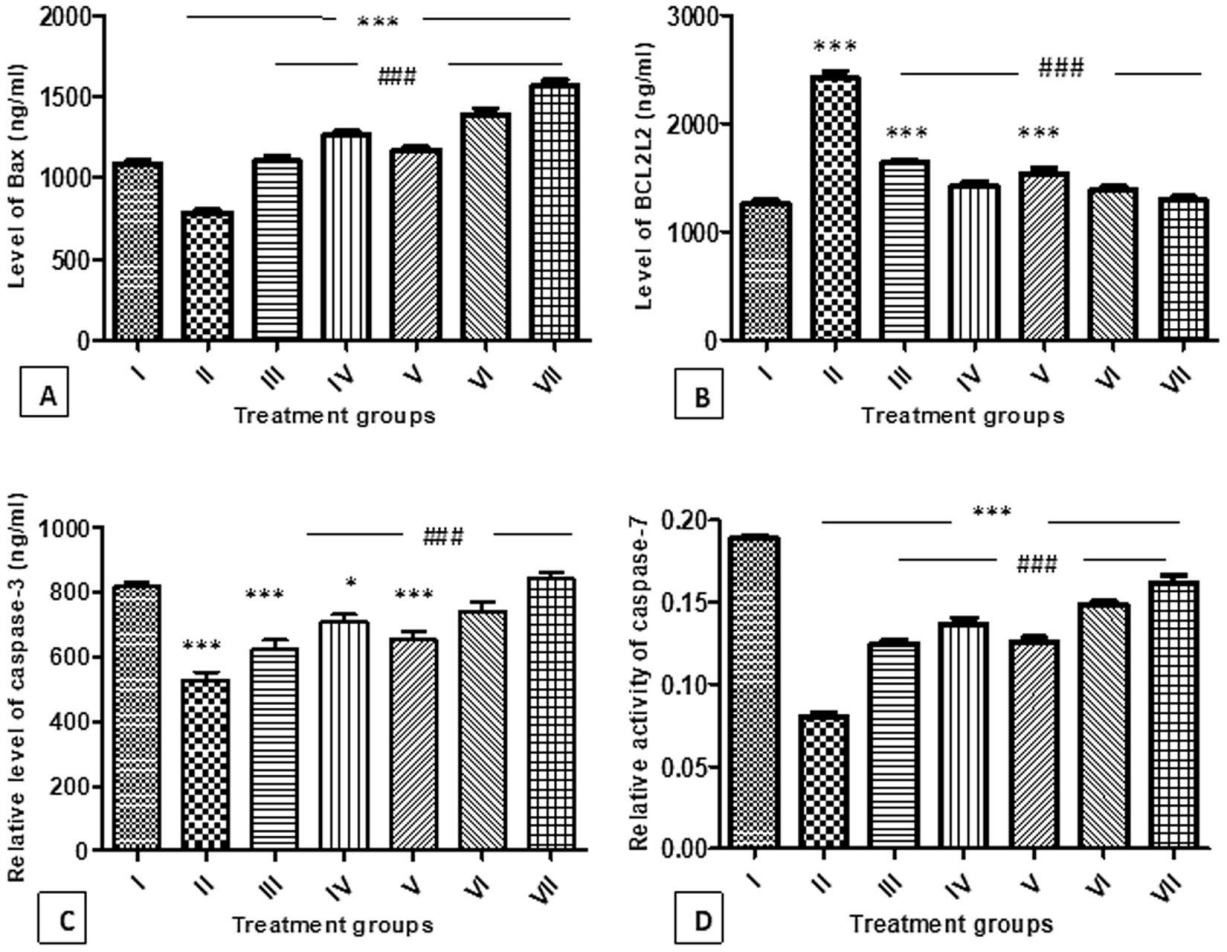
(**A**) Showing relative level of *Bax* protein in the tissue samples of different animal groups. *Indicates statistically significant from group I while #indicates statistically significant from group II. (**B**) Showing relative level of *BCL2L2* protein in the tissue samples of different animal groups. *Indicates statistically significant from group I while #indicates statistically significant from group II. (**C**) Showing relative activity of *caspase-3* in the tissue samples of different animal groups. *Indicates statistically significant from group I while #indicates statistically significant from group II. (**D**) Showing relative activity of *caspase-7* in the tissue samples of different animal groups. *Indicates statistically significant from group I while #indicates statistically significant from group II.

The level of this pro-apoptotic protein was significantly perturbed after the treatment in the animals. Group II showed a decrease in its level by 27.84% as compared to group I. Group III, IV and V exhibited an increase in its level by 40.45%, 60.78% and 49.58% with respect to the group II. However, group VI and VII demonstrated the enhancement in its level by 77.12% and 99.77% as compared to group II (Fig. 8A).

#### Effect on the level of BCL2L2 protein

The level of this anti-apoptotic protein was found elevated by 91.25% in group II as compared to group I. Group III, IV and V showed a decrease in its level by 32.41%, 41.08%, and 36.40% respectively in comparison to group II. The combination groups-VI and VII demonstrated a decline in its level by 42.47% and 46.56% (Fig. 8B).

#### Effect on caspase-3

Group II showed a decrease in its activity by 35.42% as compared to group I, while group III, IV and V demonstrated an increase in its activity by 18.63%, 34.93%, and 24.32% respectively with respect to group II. However, group VI and VII exhibited enhanced activity by 40.45% and 59.54% in comparison to group II (Fig. 8C).

#### Effect on caspase-7

Group II showed a decline in its activity by 57.45% with respect to the group I whereas group III, IV and V exhibited an increase in its activity by 55%, 70%, and 56.25% respectively as compared to group II. However, group VI and VII displayed an increase in its activity by 85% and 101.25% in comparison to group II (Fig. 8D).

#### Effect on histopathology

**Figure 9:**
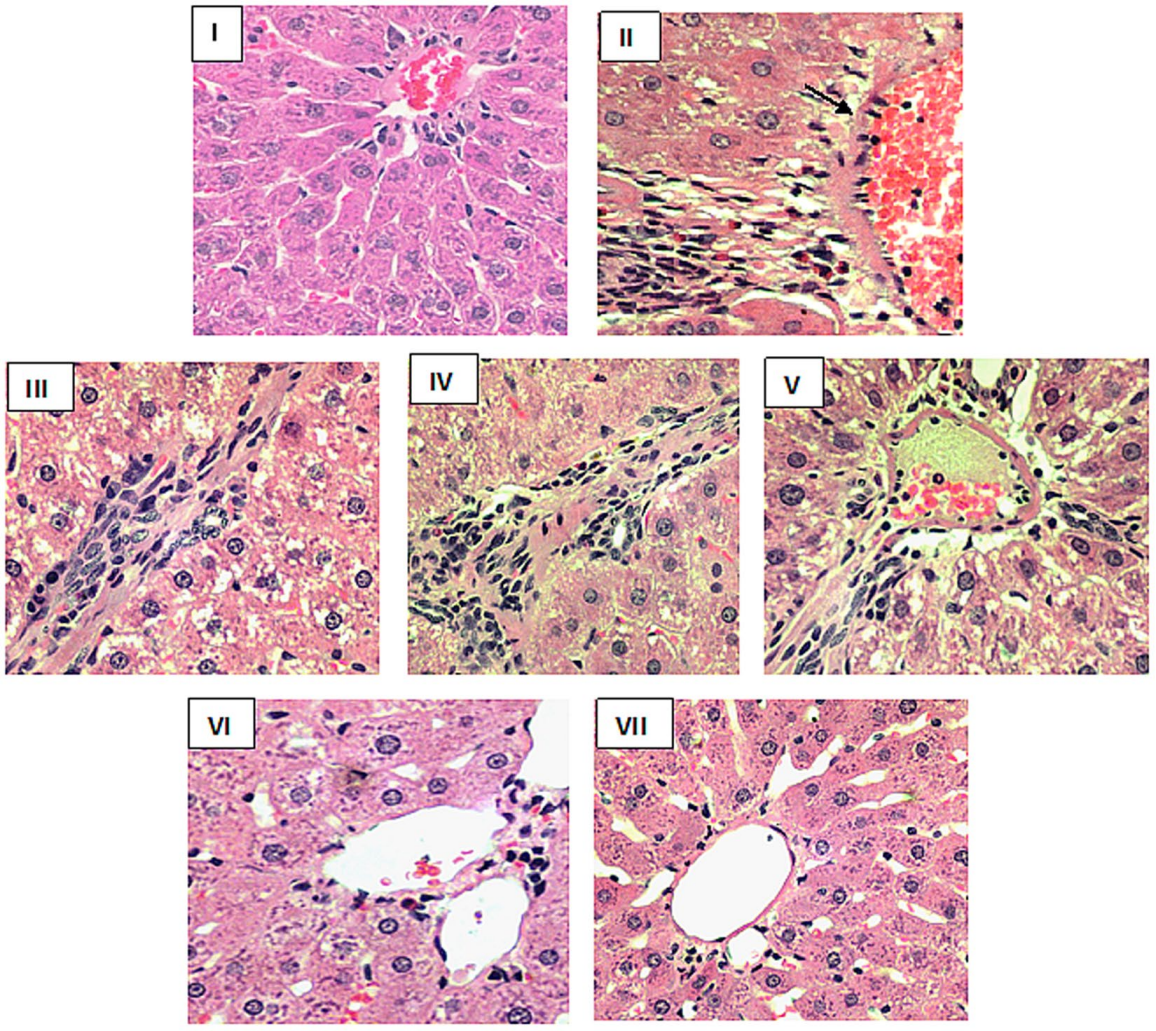
Showing histomicrographs of liver samples of various groups indicated in parentheses. All the sections were stained with Hematoxylin and Eosin and the pictures were snapped at 400X (Olympus, Japan).

The histopathology of liver tissue from the treated animals reflected marked differences among the various groups. The mild white tumor nodules/patches with the faded color of the liver in DEN-PB-treated positive control (group II) was obvious signs of hepatocarcinoma in the treated rats (pictures not shown). Control, a group I showed typical histology of the liver with the well-maintained contour of the normal hepatocytes radiating towards the central vein. The control positive, group II showed severe histological distortion in the hepatocytes with excessive vacuolation, disturbed sinusoids and inflammatory infiltration indicating partial loss of tissue in the form of fibrosis and hemorrhagic necrosis. The other groups-III and V showed moderate toxic insults in their liver histology although these features were less prevalent in group IV. The combination groups- VI and VII exhibited improvement in their histological details including decreased vacuolation and inflammatory features with less fibrosis and normal sinusoids; however, group VII showed better histological recovery than group VI with respect to the control (Fig. 9).

## Discussion

Cancer nowadays is treated by various treatment modalities including chemotherapy, radiotherapy, hormonal therapy, immunotherapy and catalytic therapy. All these modes of treatment are either effective for a short duration or accompany severe side effects during extended treatment regimens^23^. Such treatment-related toxic insults occur because there is no universal drug-target among the diverse types of cancer. Hence, the contemporary treatment modalities exert their effect on the target without differentiating between cancerous and the non-cancerous tissues leading to deleterious toxic insults. Every cell type has a set of specificity in their characteristics regarding the mechanism of drug metabolism, immune response, sensitivity towards a drug/chemical and expression of death receptors^24^. That is why any conventional treatment is not equally effective against all forms of cancer^1^. Therefore, finding common drug target for cancer treatment is one of the hot fields of research. However, the elevated copper level in cancer cells is one of the most common cellular features among the most types of cancer irrespective of their origin^25,26^. The present research work is an attempt to confirm if this common oncological characteristic can be a potential target for enhancing the antineoplastic activity of the established drugs like ITB *in vitro* as well as *in vivo*.

The cell line based *in vitro* studies in the present investigation vividly shows that Cu (II) and its chelating agent, DSF exert their differential effects on the antineoplastic efficacy of ITB in cell lines including HEK-293 and HepG2 cells as evidenced by the MTT assay. Both the agents (Cu II and DSF) were able to check the proliferation of HepG2 and HEK-293 cells. Although Cu (II) alone was also able to inhibit the proliferation of both cell lines as compared to the control, interestingly, its combination with ITB at the moderate dose demonstrated enhanced antineoplastic activity with reference to the ITB alone to a significant extent. However, the higher and lower dose of Cu (II) didn’t show any stronger antineoplastic activity alone as well as in combinations with the drug. The same pattern of improved antineoplastic activity was observed in case of ITB with a moderate dose of DSF. Additionally, the study also reveals that ITB-DSF combination exerts stronger anticancer effect than the ITB-Cu II.

As the preliminary experiments on HepG2 cells showed a better effect of the combination of the drug with the agents (Cu (II) and DSF), we furthered our study only in the same cell line. Firstly, we confirmed that exogenous Cu (II) elevates its level in the treated cells while administration of the chelating agent, DSF significantly decreases the level of the metal in the same. Moreover, the activity of PARP and p-53 was chosen as parameters to assess the induction and progression of apoptotic cell death in the treated cells. The results vividly indicate that moderate dose of both agents was able to increase PARP activity in combination with ITB. These values were quite comparable with the normal healthy liver cell line (control negative). However, ITB-DSF combination outperformed ITB-Cu (II) in both the parameters.

The tumor suppressor protein, p-53 is considered as an important cancer marker in oncological studies as it is responsible for genetic stability and suppression of tumorigenesis in the healthy cells^27^. Moreover, Surget *et al*.^28^ have documented that this protein is either mutated or diminished in most of the cancerous cells. In the present study, the cells treated with ITB-DSF combination showed the elevated activity of p-53 (S15) as compared to ITB- Cu (II) at their moderate dose. It is well-accepted among the oncologists that p-53 in the cancer cells dictate the cellular machinery for induction of apoptosis if it senses the damage in nuclear DNA beyond repair^27^. In the present study, the results also indicate that the moderate dose of Cu (II) and DSF stabilizes the protein that might help in the induction of apoptosis in the cells significantly. However, lower and higher doses of the agents were also able to trigger apoptosis moderately in the cells. The activity of PARP usually rises in the cells with massive damage to their DNA including the cancerous cells^29^. Hence, the pattern of PARP activation in the treated cells confirms the results of p-53 in the present investigation.

Furthermore, we were interested to know if the cells undergoing apoptosis involve caspase-3 or 7 pathways in the present work. Both the agents at their lower and moderate doses caused an increase in caspase-7 activity in the combination groups in a dose-dependent manner. However, the activity was found compromised when the cells were treated with the highest dose of both agents. Also, the similar pattern was observed in caspase-3 activity by the combination groups of ITB with Cu (II) and DSF. Also, lactate dehydrogenase (LDH) was chosen to assess the extent of necrosis in the treated cells in the present research work^30^. The combination of ITB with Cu (II) and DSF at the lower and highest doses demonstrated enhanced activity of the necrotic marker while the moderate dose of both the agents didn’t show any noticeable activity in the combination groups. Finally, the comet assay of the treated cells further confirmed the pattern of the previously mentioned results. The assay is believed to distinguish the cells if they undergo apoptosis and necrosis after any treatment^31,32^. Finally, the results of flow cytometry and confocal microscopy were found in agreement with the previously mentioned findings under present study.

It is noteworthy that Cu (II) exerts a different degree of antiproliferative effect on the HEK-293 and HepG2 cells as every cell line has a different level of sensitivity and behavior towards a particular agent/drug^33^. Furthermore, the manner of transport and intracellular mechanism of action of a drug also differ from one cell line to another that is also attributive in exerting its differential effect on the different cell lines^34^. Additionally, the moderate amount of Cu (II) has been reported in elevation of the oxidative stress in the cells that might push the target cells to undergo either apoptosis or the necrosis^35–37^. The low dose of copper causes a lesser degree of DNA damage in the target cells that can be repaired by the adaptive response in the cells that consequently retrograde the antineoplastic activity of ITB as evidenced in the present study^38^. However, the moderate dose of Cu (II) acts as a pro-oxidant, and that can facilitate the cellular redox status in the target cells to the optimum required for induction of the programmed cell death. Besides, this dose of the metal may aid to the endogenous copper binding to the chromatins (in the packaged nuclear DNA with proteins) of the healthy cells stabilizing their genome as well as prompting them for cell division^39^. However, the copper metabolism in the cancer cells is vastly altered, so the stabilization of their nuclear DNA by supplemented Cu (II) might not be sufficient in the present work. These conditions may further engage apoptotic proteins like p-53 and PARP either for repair of the damaged DNA or induction of apoptosis. Also, all these features of Cu (II) at the moderate dose might enhance the apoptosis-inducing propensity of ITB as shown by the combination groups in comparison to the drug alone. However, the higher dose of copper in the treated cells elevates the oxidative stress over the threshold level that can pronounce its pro-oxidant activity damaging the nuclear DNA beyond repair leading to aponecrosis and necrosis. The current study proves these notions. The pro-oxidant nature of Cu (II) can generate various ROS that can invade multiple cellular organelles and components including nuclear DNA and even *p-53* gene^38,40^. Additionally, the metal at a higher concentration can affect various genes including those involved in cell cycle, tumorigenesis and cell death that are profoundly functional to the cellular redox status and energetics. In HepG2 cells, the copper can modulate the expression of genes like HNF-α and ATF3 that further can affect p-53 gene^41^. In the present study, ITB and copper might exert a different effect on all these target genes. The elevated Cu (II)-induced ROS can either modify p-53 or can modulate it in the presence of ITB differently or, the cross-talk among these genes affected by the drug with a higher concentration of the metal compound. This complex interaction between the drug and test chemical(s) might assist in the cell survival instead of apoptosis as evidenced by the results of the present study. The combination groups exert their antineoplastic activity synergistically in the present study. Nevertheless, the interaction between ITB and Cu (II) itself cannot be ruled out since there was a prominent compromise in the antineoplastic activity of the drug with the higher dose of the metal compound.

In addition, these *in vitro* findings are well supported by the *in vivo* results under the present study. The chemically induced hepatocarcinoma in the rats was confirmed by excessive elevation in their liver function markers (ALT, AST, bilirubin, and GGT) and extensive tissue damage under histopathological assessment in the control positive group^42,43^. However, treatment of those rats with the standardized dose of Cu (II), DSF and ITB showed mild to moderate improvement in most of the parameters. Intriguingly, the combination of DSF and Cu (II) with ITB demonstrated significant improvement as compared to control positive. Furthermore, the suppression of the level of *Bax* (an anti-apoptotic protein) and overexpression of *BCL2L2* (a pro-apoptotic protein) in the combination groups further consolidated the biochemical analysis. Moreover, the heightened activity of caspase-3 and 7 also confirms that combinations of the agents with the drug facilitate apoptosis induction in the target organ, liver. These results were found in coherence with the histopathological evaluation of the same groups. These findings indicate that the test agents (DSF and Cu II) enhance the antineoplastic activity of the drug, ITB to a significant extent. Hence, both the *in vitro* and *in vivo* studies seem in agreement with the hypothesis proposed in the present investigation. The *in vivo* study also revealed that ITB-DSF combination showed the better synergistic effect on the anticancer activity of ITB as compared to the ITB-Cu (II) combination up to a significant level in most of the studied parameters.

It is important to note that the cancer induction by DEN-PB combination takes at least 20 weeks for full-blown hepatocarcinoma^14^. Cancer-induced by this combination of chemicals occurs in three stages: inflammatory stage (2–6 weeks); tissue-fibrosis stage (8–12 weeks) and full-blown cancer (14–20 weeks)^43^. Hence, cancer induced in the present study seems to be from the fibrosis stage rather than the full blown. Thus, the proposed combinations of the drug with the agents might show the pronounced antineoplastic action in the present investigation that needs to be tested in full-blown cancer models (study in progress). However, excessive elevation in liver function markers along with total bilirubin and alpha-fetoprotein, a prominent tumor marker (data not shown) confirms severe liver toxicity and damage akin to hepatocarcinoma in the present study.

Furthermore, DSF was included to confirm the role of elevated Cu (II) in the maintenance of cancer cells. Intriguingly, DSF in the present study exhibits stronger adjuvant property than Cu (II) to the antineoplastic efficacy of ITB. Lots of literature implies that DSF is a potential anticancer agent itself as it counters the elevated level of Cu (II) towards the normal^44^. Hence, DSF perhaps sensitizes the cancer cells against the chemotherapeutic agents by altering their cellular stress and compromising the stability of the nuclear chromatin DNA in the absence of Cu (II). Recently, Duan *et al*.^45^ have reported that DSF makes a complex with intracellular copper leading to the destruction of cancer stem cells and clusters of cancer cells by scaling up the oxidative stress level. The present research entails that adjuvant property of DSF towards the antineoplastic activity of ITB is a dose-dependent up to a certain level. Furthermore, Dastjerdi *et al*.^46^ have documented that DSF at moderate dose exerts an inhibitory effect on NF-κB pathway, matrix metalloproteinases (MMP-2, MMP-9) and topoisomerase I and II activities concomitant with inhibition of metastasis and angiogenesis. Also, it changes the intracellular superoxide levels with decreased mitochondrial membrane polarization resulting into induction of apoptosis as well as halting of proteasome activity as observed in cell lines based studies^47^. All these cellular consequences post-DSF treatment seems in control if the dose is moderate. On the contrary, a higher dose of DSF might exhaust the endogenous copper and other essential divalent metals that might disrupt the balance between cellular energetics and metabolism that can further dictate the cells either to undergo incomplete apoptosis, aponecrosis or necrosis depending on the susceptibility of the cells and their concurrent microenvironment.

Alternatively, elevation of Cu (II) in the cancer cells may be one of the cellular response strategies to handle the burden of tumorigenesis that might be required for increasing angiogenesis as well as enhancing the stability of nuclear chromatin as a means of chemo-resistance^48^. In the same league, the elevated copper level might instruct the cellular machinery to scan through the nuclear DNA for any damage. As per the status of the nuclear integrity, the cell either halts cell cycle progression until the repair of the damaged DNA. Nevertheless, if the cell senses that the DNA damage incurred is beyond repair or highly uneconomic (regarding NADH/ATP), then it might activate programmed cell death via activation of caspase-7 and 3. In that case, other critical pro-apoptotic markers including p-53 and PARP also assist in the execution of programmed cell death. The present study also reveals that the moderate dose of DSF and Cu alone as well as in the combination groups with ITB is the best-suited treatment regimen to engage these significant facilitators of apoptosis. However, higher and lower from the threshold dose might under-activate or over-activate the same factors that might consequently either hinder apoptosis progression or end up as incomplete apoptosis or even aponecrosis. Nevertheless, these extreme doses of the Cu (II) And DSF might trigger necrosis that might be attributive in enhancing cell proliferation even at the higher dose of both the agents alone and also with the drug. Similar findings reported on chelating agents including DSF further confirms our results^49–51^. Interestingly, DSF in the present study synergizes the antineoplastic effect of ITB by countering the excess endogenous copper in the cancer cells that possibly reverse all the prevailing chemo- resisting macro- and micro-environment. As a consequence, the tumor cells recruit cellular and immune responses that might pave the pathways for apoptosis induction. It is also possible that higher copper dose might disrupt/destruct the copper transporters that are also drug carriers across the cells from their *milieu* leading to compromised drug delivery into the cells. On the contrary, the moderate dose of DSF might chelate extracellular copper keeping the drug transporter protein(s) for efficient drug delivery across the target cells. All these factors might weigh in favor of DSF in increasing the antineoplastic activity of ITB as compared to Cu (II).

## Materials and Methods

### Chemicals and reagents

All the test chemicals- Copper chloride (CuCl_2_), Imatinib (ITB) and Disulfiram (DSF) were purchased from Sigma-Aldrich, St. Louis, MO, USA. The reagents for cell culture including DMEM, RPMI-1640, antibiotic-antimycotic solution, trypsin solution, fetal bovine serum (FBS), phosphate buffered saline (PBS) were purchased from either Gibco, USA or, Sigma-Aldrich, St. Louis, MO, USA.

### Methods

The present study was conducted on four cell lines - MCF-7, MDA-MB 231 (both breast cancer cell lines), HEK 293 (Kidney cancer cell line) and HepG2 (Liver cancer cell lines). All the cell lines were purchased from American type cell culture (ATCC, USA). In this study, the cell lines were treated with three test chemicals- copper chloride (CuCl_2_ or Cu II), imatinib (ITB) and disulfiram (DSF). For the detailed study in the present work, HepG2 cells were divided into following thirteen groups:

Group I: Control positive (HepG2 without any treatment)

Group II: CuCl_2_ (7.43 mM).

Group III: CuCl_2_ (14.86 mM).

Group IV: ITB (1.69 mM).

Group V: ITB (3.38 mM).

Group VI: ITB (1.69 mM) + CuCl_2_ (3.71 mM).

Group VII: ITB (1.69 mM) + CuCl_2_ (7.43 mM).

Group VIII: ITB (1.69 mM) + CuCl_2_ (14.86 mM).

Group IX: DSF (1.18 mM).

Group X: DSF (3.37 mM).

Group XI: ITB (1.69 mM) + DSF (1.18 mM).

Group XII: ITB (1.69 mM) + DSF (3.37 mM).

Group XIII: ITB (1.69 mM) + DSF (6.74 mM).

Group XIV: Control negative (healthy liver cell line).

All the cell lines were cultured separately in complete media containing DMEM and RPMI-1640 medium including 10% fetal bovine serum (FBS, Sigma-Aldrich, USA), 100 IU/ml penicillin and 100 mg/ml streptomycin (Gibco, USA). The cells were provided a humidified CO_2_ incubator set at CO_2_: air ratio as 5%: 95% at 37 °C by the standard growing method. The cultured cells were observed and snapped under inverted Microscope (Nikon, Japan) for assessment of their morphology and cellular integrity. The medium was changed after every 2–3 days. All these cell lines based experiments were conducted under a sterile condition under Biosafety Cabinet (Labmed, Korea). After incubation for 5–6 days, the cells were observed under the microscope if they have reached 90% of confluence. Once, they were grown enough; they were sub-cultured (every 2–3 days) after detaching with 0.25% trypsin/27 mm EDTA (5 min, 37 °C).

#### Preparation of test chemicals (drugs)

All the test chemicals- Copper chloride (Cu II), Imatinib (ITB) and Disulfiram (DSF) were purchased from Sigma-Aldrich, USA.

All of the chemicals for their treatment with cell lines were dissolved in PBS to prepare in 10 ml stock solution (concentration of 1 µg/µl) for each. Then, the test compounds were treated at following four different volume from the stock (11.2 µl, 25 µl, 50 µl and 100 µl) with 100 µl of the cell suspension (~50,000 cells) pre-seeded in Elisa plate (Corning, USA).

### MTT assay

The assay was conducted by commercially available kit (Vybrant MTT Cell Proliferation Kit) in all four cell lines. All the test chemicals were pippeted on pre-seeded cell lines (90% confluence) in Elisa plates and were incubated in CO_2_ incubator programmed as 5% CO_2_: 95% air at 37 °C for 48 h. After this, MTT (50 µl) was added to each well and were allowed to incubate for three h. After completion of incubation, the solubilization solution (50 µl) was added and waited for 15 minutes. Finally, the plates were read at 630 nm in Elisa plate reader (Anthos 2020, UK).

### Measurement of intracellular copper

HepG2 Cells (1 × 10^6^) were plated with serum-free media in tissue culture Petri dishes (Nunc, Thermo Fischer Scientific, USA). After their full-blown growth on the day, the media was replaced by 1% FCS medium supplemented with the test chemicals as per the treatment groups. After 24 h of treatment, the cell medium was stored separately followed by washing of the cell monolayer with HBSS (calcium-free). Furthermore, the monolayer was treated with trypsin/EDTA for the living cells as well as scraped to get the fixed cells. The total cells were dissolved in PBS and centrifuged at 800 × g^51^. The pellet was taken as the sample for copper measurement by commercially available kit (Sigma- Aldrich, USA) for respective groups.

### p-53 assay

This assay was conducted by the commercial kit (Cat. No. CBEL-p53–1, Ray Biotech, USA) in HepG2 cell line. For this assay, the procedure booklet with the kit was employed step by step with few modifications. Almost 30,000 cells were seeded in each well which was allowed to grow in the medium for overnight. The treatment chemicals were added according to manufacturer’s instructions. After that, 100 µl of Fixing Solution was added to each well and incubated for 20 minutes at room temperature. Further, 200 µl of prepared Quenching Buffer (1X) was allowed to incubate for 20 minutes at room temperature. Then, 200 µl of prepared Blocking Buffer (1X) was added and incubated for 1 hour at 37 °C. 50 µl of prepared 1X primary antibody was pipetted into each well and incubated for 2 hours at room temperature. 50 µl of prepared HRP Conjugated secondary antibody (1X) was added and incubated for 1 hour at room temperature. Followed by, 100 µl TMB Substrate was added and incubated 30 minutes at room temperature. Lastly, 50 µl Stop Solution was pipetted into each well. Then the Elisa plate was read at 450 nm immediately.

### PARP assay

This assay was conducted only in HepG2 cell line by commercially available kit (Cat. No. 4684–096-K, Trevigen, USA). The protocol associated with the kit was employed for the assay with slight modifications. On Day 0, treated actively growing cells were seeded in fresh medium/well in a 96 well flat-bottom plate for adherent cells with separate aside triplicate wells as controls (non-treated). Then on Day 1, 1 µl of 10 mM Etoposide was added followed by addition of treatment agents in triplicate wells for 50 µM final concentration that was incubated at 37 °C/5% CO_2_ for 6-h time points. Further, 1 µl of 10 mM Etoposide in triplicate wells were set up for the 4 h, 2 h and remaining time points. Rest all the steps under the protocol were followed and the plate was read at 450 nm (Biochrom, Anthos2020, UK).

### Assay of Lactate Dehydrogenase (LDH)

Recently, LDH has been reported to be a reliable marker for necrosis^30^. The assay was conducted by commercially available kit (Cat. No. ab102526, Abcam, UK) by Elisa using microplate reader (Biochrom, Anthos2020, UK).

### Assay of Caspase-7

This assay was conducted with the commercially available kit (Cat. No. ab157404, Abcam, UK,) on the HepG2 cell lysate after the treatment with the test compounds. The instructions booklet with the kit was followed for the assay using a fluorescent microplate reader (Bio-Rad, USA).

### Assay of Caspase-3

The assay was conducted on treated HepG2 cells by commercially available kit (Cat. No. # SC-4263, Santa Biotechnology, INC., USA) following the manufacturer’s instructions using a fluorescent microplate reader (Bio-Rad, USA).

### Comet Assay (Single cell gel electrophoresis)

This assay is considered one of the reliable genotoxicity measuring techniques to assess damage in nuclear DNA in the target cells. In the present study, this experiment was performed with the method developed by Singh *et al*.^52^ with few adjustments^31,32,53^. They full grown HepG2 cells were subjected to the test chemicals for three hours in different Petri-dishes (60 × 15 mm, Greiner) in the suitable media and conditions as mentioned in Methods 2.2. Rest all steps were followed by the method explained by Shaker *et al*. and Olive *et al*.^31,53^. The migration patterns of nuclear DNA of 100 cells for each group was assessed by an upright fluorescent microscope (Leica DM2500, Germany) attached with a digital CCD camera (Andor Zyla 5.5, UK). The measurement of tail length with imaging was conducted by Komet 5.5 image analysis software (Andor, UK).

### Confocal microscopy

The treated cells were stained with Propidium iodide, and Alexa Fluor 488 on poly L-lysine coated microscope slides followed by placing with coverslips. The slides were analyzed by confocal microscopy (Carl Zeiss, Germany) with a Zeiss LSM 5 EXCITER confocal laser-scanning module and the images were saved (100X) after analyzing with the instrument’s software.

### Assessment of cell events of the treated cells by flow cytometry (FACS)

The evaluation of apoptosis and necrosis was conducted in the important treatment groups by Tali Apoptosis kit- Annexin V Alexa Fluor 488^™^ (Thermo Fisher Scientific, USA). The whole experiment was done as per the provided kit manual. The samples were analyzed in a flow cytometer (Beckman Coulter, FC500, Brea, CA) using forward scatter and side scatter with a flow rate of the machine maintained set on 30 µl/s.

### *In vivo* studies

After getting good results *in vitro* studies, we wanted to check our hypothesis *in vivo*.

#### Animal husbandry

Seventy male Swiss Albino rats (120 ± 20 g, 3–4 months old) were purchased from Central Animal House (Department of Pharmacy, King Saud University, Riyadh). All the rodents were allowed to acclimatize for ten days in standard conditions in the Departmental Animal house (Department of Zoology, King Saud University, Riyadh). They were reared under ethically approved conditions of optimum temperature (22 ± 3 °C) and appropriate humidity with 12 h day: night cycle on a standard pellet diet and fresh water *ad labium* in sufficiently large cages. All rearing and treatment procedure involving animals were conducted under Institutional ethical rules cleared by Institutional Ethical committee of King Saud University, Riyadh.

#### Treatment with the test chemicals

The experimental animals were randomly divided into seven groups (n = 10). The group I was kept as a control negative (CN-) treated with 1 ml of saline. Remaining all the groups (II to VII) were treated with diethylnitrosamine (100 mg/kg body weight once a week for a month) and phenobarbital at the dose of 0.05% (w/v) in the drinking water in all groups (except the CN-) for two months. Both the chemicals were chosen as a pair of carcinogen (tumor inducer) and a promoter for induction and development of hepatocellular carcinoma in the rats^54,55^. After two months, group III, IV and V were treated with copper chloride (Cu), imatinib (ITB) and disulfiram (DSF) at the standardized dose of 25, 35 and 50 mg/kg. Moreover, group VI was administered with ITB and Cu while group VII was injected with ITB and DSF at their respective doses. All these groups (III to VII) were put on freshwater ad labium during the remaining treatment. Dosing of the test chemicals was based on the treatment strategy of Hassan *et al*. (2012)^23^.

#### Confirmation of cancer induction

The rats were monitored for their body weight and any obvious visible alteration throughout the treatment. After completion of the first month of cancer induction, blood was withdrawn from a retro-orbital region of two members of each group to check liver function tests [aspartate transaminase (AST), alanine transaminase (ALT), γ-glutamyl transferase (GGT)] in serum samples to confirm the occurrence of hepatocarcinoma^54^. Furthermore, liver specimens were removed from two rats in each of group for histopathological analysis^54^. Furthermore, Alfa-fetoprotein (AFP), an important tumor marker for confirmation of tumorigenesis, was assessed by a commercial kit (data not shown).

#### Sample collection

During the treatment with test chemicals, 8 rats died among the cancer-induced animals. Remaining rats were sacrificed on a single day, and their liver was washed in PBS (pH 7.4) and stored at −80 ^°^C (Eppendorf, UK). Besides, blood was withdrawn in vacuum tubes (with anticoagulant), and the serum was stored at −80 °C (Eppendorf, UK) after centrifugation at 1200 × g ((Eppendorf, UK).

The liver of the all groups were divided into two parts: the first part for histopathology while second part for biochemical and molecular studies.

#### Biochemical studies

The organ from each animal was homogenized (Ika, USA) in potassium phosphate buffer (0.1 M, pH 7.36) followed by their centrifugation at 3000 × *g* for 10 min (Eppendorf, Germany). The serum was collected from the blood as well. Thus the samples with proper labeling were stored at −80 °C till their biochemical analysis.

### Assessment of target organ toxicity in serum

Measurement of aspartate transaminase (AST) and alanine transaminase (ALT) was conducted by the commercial kits under manufacturer’s instructions (QCA, Spain). Moreover, measurement of conjugated bilirubin and γ-glutamyl transpeptidase (GGT) were conducted by the commercial kits (BioVision, USA) under manufacturer’s instructions.

#### Assessment of apoptotic markers in liver samples

The level of apoptotic markers including Bax and BCL2L2 in the samples was measured by commercial kits following the manufacturer’s instructions (Thermo Fisher scientific company, USA).

#### Assessment of mode of cell death

The evaluation of mode of cell events in the target tissues was conducted by measurement of the activity of caspase-3 and caspase-7 by the commercial kits (Thermo Fisher scientific company, USA) as per the kit protocol.

#### Histopathology of the target organs

The liver samples of treated groups of the rats were be stored in 8% formalin for immersion fixation for their histopathology studies. For their paraffin embedding, 10 × 5 × 3 mm sized tissue blocks of the organ were prepared. Their sections of 7 micrometers (µm) thickness were cut with rotary microtome followed by staining with Hematoxylin and Eosin stain. The sections were observed under a light microscope, and their photomicrographs were captured at the magnification of 400 X (Olympus, Japan).

### Statistical analysis

All the experimental data were represented as Mean ± SEM analyzed by GraphPad Prism 5 software. The data analysis was conducted by one-way ANOVA with Tukey’s post-hoc selecting p-value < 0.05 provided with the software. The number of asterisk mark* and #indicates the extent of statistically different from the respective positive control (group I) and ITB treated (group IV) values *in vitro* studies. However,* and #indicates significantly different from negative control (group I) and positive control (group II) *in vivo* studies. Minor fluctuations were found on repetition of the experiments as indicated in the statistical analysis.

### Ethical clearance

All the study design and experiments based on animals were strictly conducted going by the guidelines for the care and use of experimental animals by the Committee for the Purpose of Control and Supervision of Experiments on Animals (CPCSEA) and the National Institutes of Health (NIH). All animal-based experiments were approved by the Animal Ethics Committee of the Zoology Department in the College of Science at King Saud University, Riyadh, KSA.

## Conclusion

The present investigation demonstrates that level of copper in cancer cells is critical in determining the efficacy of chemotherapy. Any agent/adjuvant that can bring the endogenous copper level to a threshold level orchestrating the cellular and molecular predilection for apoptosis induction, can qualify to be an effective anticancer drug. In the present work, cupric chloride and disulfiram enhance the antineoplastic activity of imatinib to a significant extent *in vitro* as well as *in vivo*. However, further studies are necessary for understanding the exact mechanism and effects of the proposed adjuvants with ITB.
